# Supplementary material for: Meta-analysis reveals that phenotypic plasticity and divergent selection promote reproductive isolation during incipient speciation
Source: Nat Ecol Evol. 2025 May 7;9(5):833–44. doi: 10.1038/s41559-025-02687-7 (PMC12066359; doi:10.1038/s41559-025-02687-7)
Supplement: Supplementary file 1 — Supplementary Figs. 1–5 and Tables 1–5. [file 41559_2025_2687_MOESM1_ESM.pdf]

nature ecology & evolution

Supplementary information https://doi.org/10.1038/s41559-025-02687-7

Meta-analysis reveals that phenotypic plasticity and divergent selection promote reproductive isolation during incipient speciation

In the format provided by the authors and unedited

Supplementary Figures 1–5

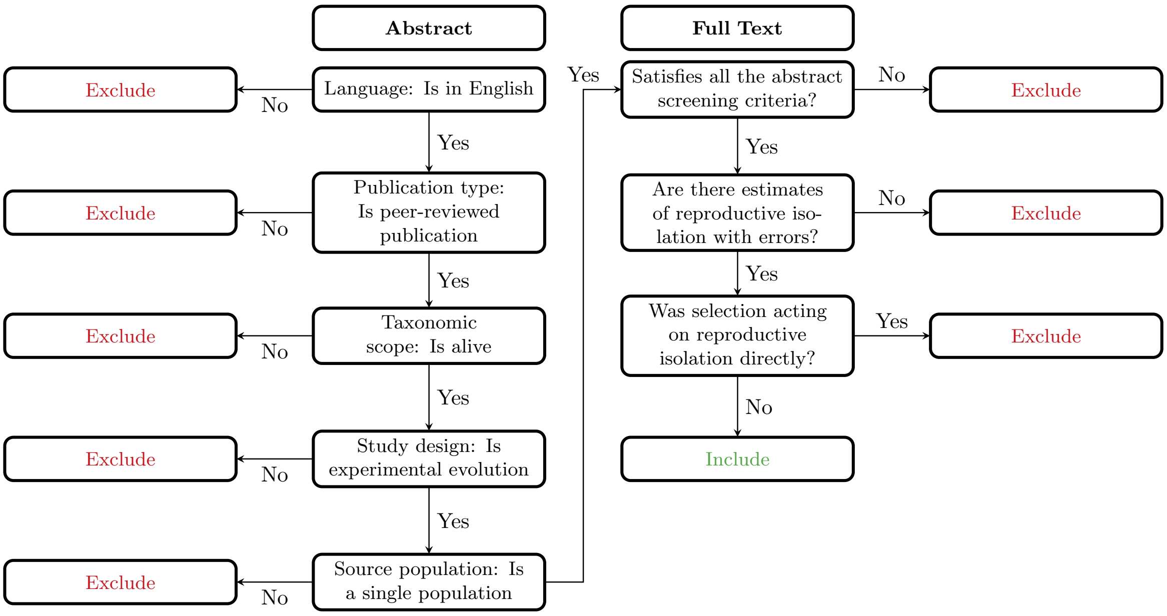

Supplementary Figure 1 | Decision tree for title and abstract screening, and full text screening.

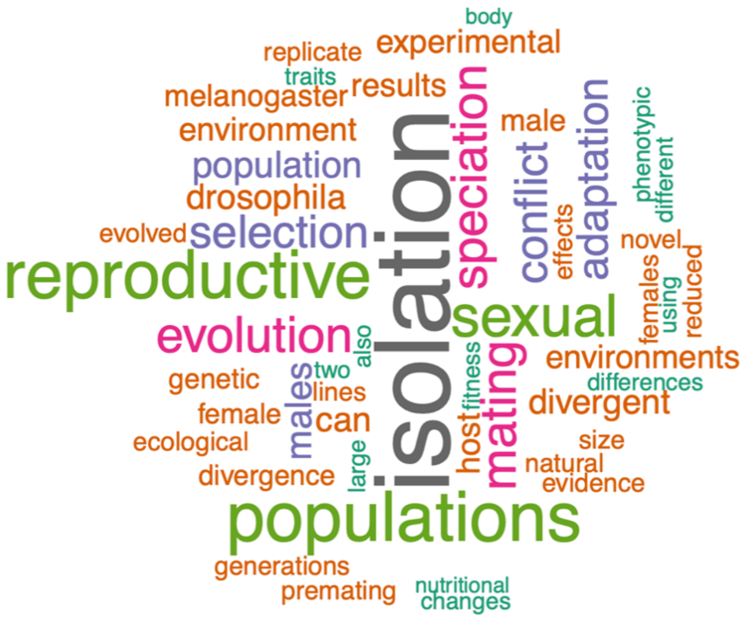

Supplementary Figure 2 | Word cloud formed from the title and abstracts of the 15 benchmarking papers (Supplementary Table 1). This guided our construction of search strings.

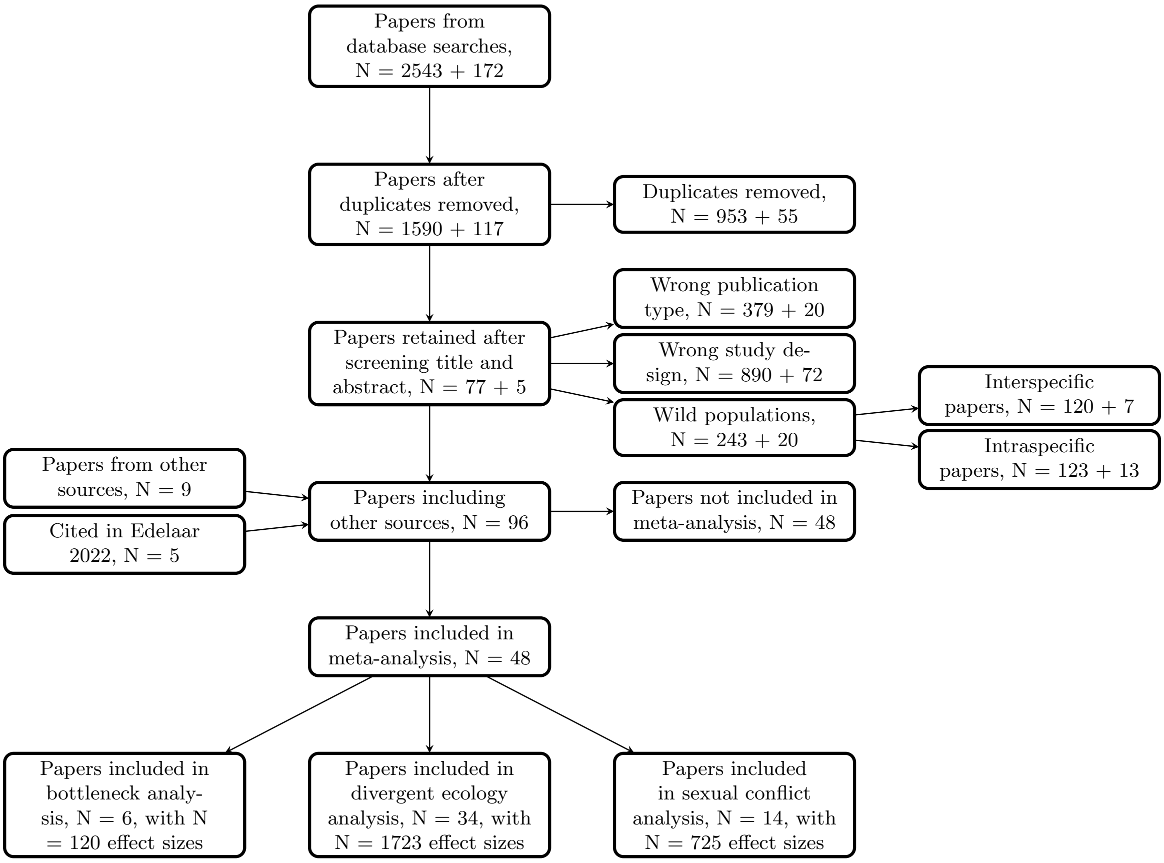

Supplementary Figure 3 | PRISMA flow chart. The two numbers in each block are from the two searches (the first in March 2021 at Lund University and the other in June 2023 at Bangor University. This manuscript covers only the results extracted from the middle block at the bottom "*Papers included in the divergent ecology analysis*". The blocks either side are included for completeness.

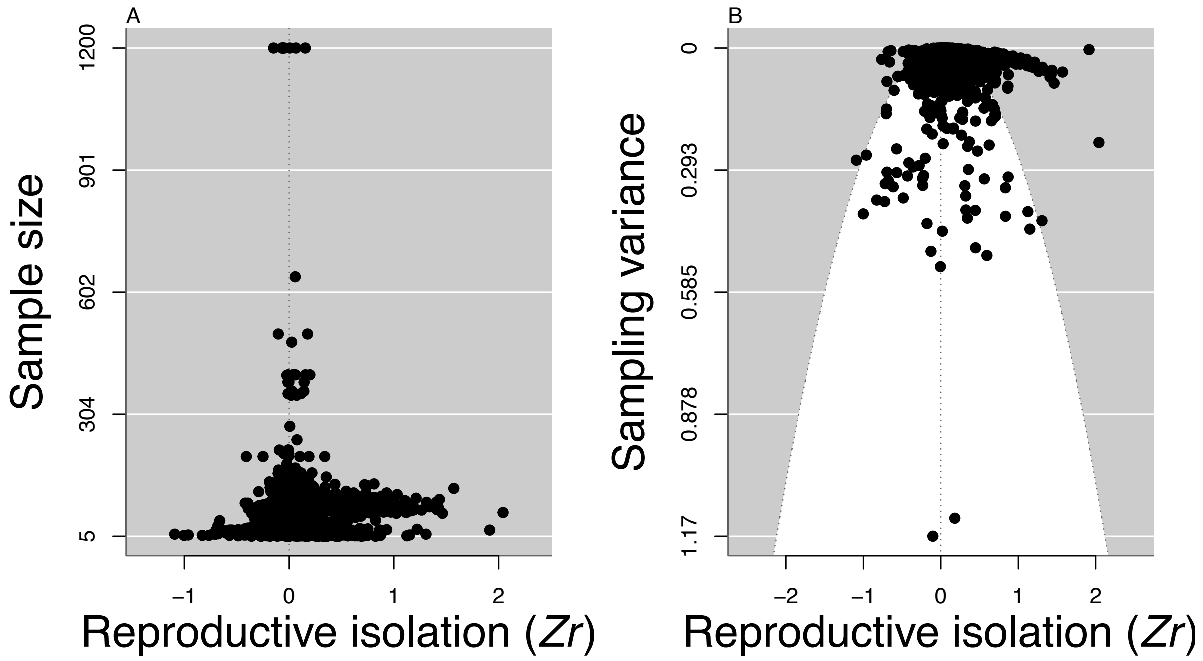

Supplementary Figure 4 | Meta-analytic funnel plots. The relationship between reproductive isolation and sample size (A) and reproductive isolation and sampling variance (B). Both plots show significant bias with skew to the right.

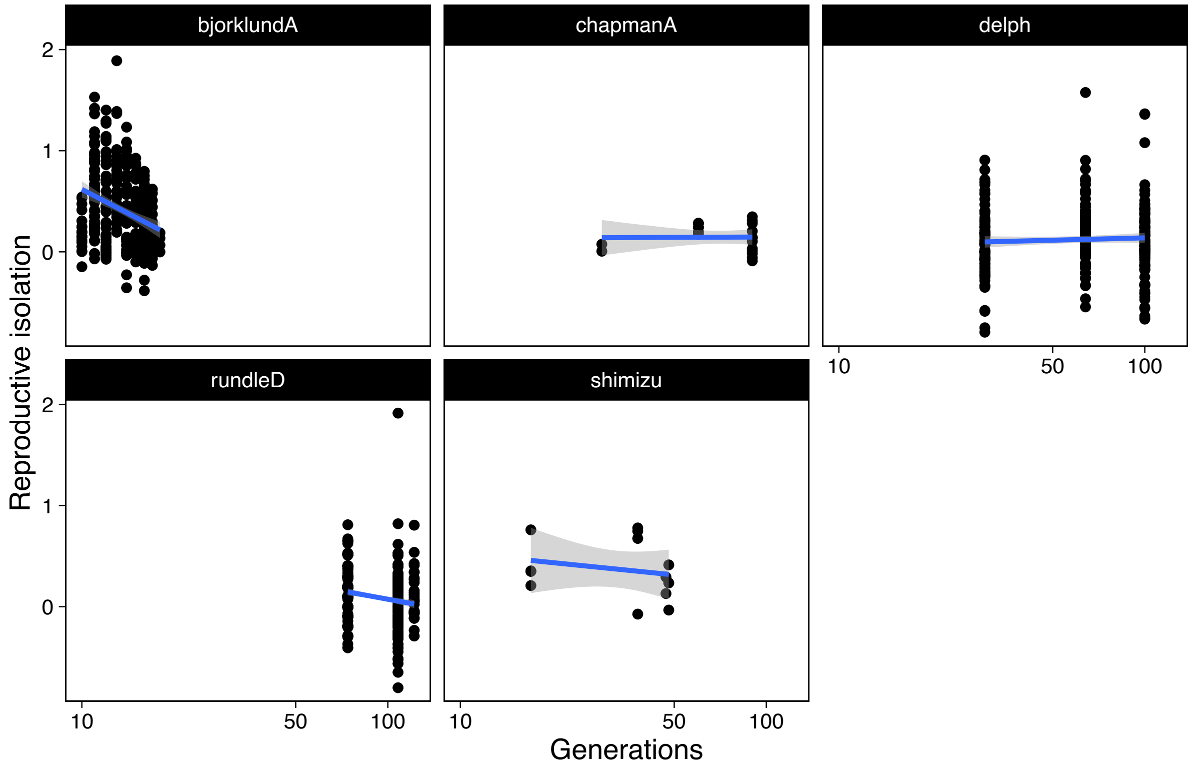

Supplementary Figure 5 | Studies that have estimated reproductive isolation in the same populations in more than one generation. Lines of best fit with standard errors as the grey shaded area are shown to illustrate the overall patterns of reproductive isolation over time for each study as they are estimated without any reference to the covariance between populations used more than once to produce reproductive isolation estimates, or without reference to precision of the data. The papers that contributed data to each panel are: bjorklundA[100,101]; chapmanA[89,95]; delph[31]; rundleD[61]; and shimizu[93].

Supplementary Tables 1–5

Supplementary Table 1 | List of benchmarking papers used for our meta-analysis search. All papers were found in our literature search.

Authors	Year	Paper title	Journal
Rice	1996	Sexually antagonistic male adaptation triggered by experimental arrest of female evolution	*Nature* 381, 232
Galiana et al.	1996	Postmating isolation analysis in founder-flush experimental populations of *Drosophila pseudoobscura*	*Evolution* 50, 941
Rundle	2003	Divergent environments and population bottlenecks fail to generate premating isolation in *Drosophila pseudoobscura*	*Evolution* 57, 2557
Martin & Hosken	2003	The evolution of reproductive isolation through sexual conflict	*Nature* 423, 979
Wigby & Chapman	2006	No evidence that experimental manipulation of sexual conflict drives premating reproductive isolation in *Drosophila melanogaster*	*J. Evol. Biol.* 19, 1033
Dettman et al.	2007	Incipient speciation by divergent adaptation and antagonistic epistasis in yeast	*Nature* 447, 585
Hosken et al.	2009	Sexual conflict and reproductive isolation in flies	*Biology Letters* 5, 697
Kwan & Rundle	2010	Adaptation to desiccation fails to generate pre- and post-mating isolation in replicate *Drosophila melanogaster* laboratory populations	*Evolution* 64, 710
Ghosh & Joshi	2012	Evolution of reproductive isolation as a by-product of divergent life-history evolution in laboratory populations of *Drosophila melanogaster*	*Ecology & Evolution* 2, 3214
Matute	2013	The role of founder effects on the evolution of reproductive isolation	*J. Evol. Biol.* 26, 2299
Arbuthnott et al.	2014	The ecology of sexual conflict: ecologically dependent parallel evolution of male harm and female resistance in *Drosophila melanogaster*	*Ecology Letters* 17, 221
Stojkovic et al.	2014	Host-shift effects on mating behaviour and incipient pre-mating isolation in seed beetle	*Behavioural Ecology* 25, 553
Castillo et al.	2015	Experimental evolution: assortative mating and sexual selection, independent of local adaptation, lead to reproductive isolation in the nematode *Caenorhabditis remanei*	*Evolution* 69, 3141
Villa et al.	2019	Rapid experimental evolution of reproductive isolation from a single natural population	*PNAS* 116, 13440
Nash et al.	2019	Mate choice and gene expression signatures associated with nutritional adaptation in the medfly (*Ceratitis capitata*)	*Scientific Reports* 9, 1

Supplementary Table 2 | Databases covered by Web of Science at the two universities where searches were performed. The Scopus search only searched the Scopus database.

Institution	Databases covered by Web of Science
Lund University	Web of Science Core collection, BIOSIS Previews, CABI, Conference Proceedings, Emerging Sources Citation Index, FSTA, Korean Journal Database, MEDLINE, Russian Citation Index, SciELO Citation Index, Zoological Record
Bangor University	Web of Science Core Collection, Biological Abstracts, BIOSIS Previews, Zoological Record, Current Contents Connect

Supplementary Table 3 | The list of papers included in the divergent selection dataset, with details of the species used, the selection imposed, when (in generations) reproductive isolation was estimated, and the amount of data each has contributed to the dataset. Two pairs of papers (indicated with the † and ‡ symbols) estimate reproductive isolation on the same populations. Full references numbers refer to the main reference list of the paper.

Paper	Species	Source of selection	Generations	Number of effect sizes
Arbuthnott et al.[82]	*Drosophila melanogaster*	Diet (ethanol or cadmium)	219	56
Barerra et al.[67]	*Drosophila melanogaster*	Diet (cornstarch-yeast and heat shock or cornmeal and NaCl at constant temperature)	74, 108, 122	448
Belkina et al.[83]	*Drosophila melanogaster*	Diet (standard or starch or high salt)	58, 103	17
Bérénos et al.[84]	*Tribolium castaneum*	Parasite (coevolution with *Nosema whitei* or no parasite)	16	2
Boake et al.[85]	*Drosophila melanogaster*	Pesticide (DDT)	1100	1
Bordas et al.[86]	*Gallus gallus*	Artificial selection on residual feed consumption	16	3
Castillo et al.[31]	*Caenorhabditis remanei*	Diet (*Escherichia coli* or *Bacillus subtilis*)	30, 64, 100	331
Dargent et al.[87]	*Poecilia reticulata*	Ponds with or without predators	8, 12	6
Dettman et al.[88]	*Saccharomyces cerevisiae*	Diet (high salinity or low glucose)	500	18
Dettman et al.[89]	*Neurospora crassa*	Diet/temperature (high salinity and high temperature or low salinity and low temperature)	1362, 1589	4
Easty et al.[90]	*Poecilia reticulata*	Ponds with or without predators	13, 26	2
Falk et al.[91]	*Tribolium castaneum*	Diet (wheat or corn flour)	43	5
Fry[92]	*Tetranychus urticae*	Diet (host plant: tomato or bean)	10	1
Fry[25]	*Drosophila melanogaster*	Diet (ethanol or regular)	120	6
Ghosh & Joshi[93]	*Drosophila melanogaster*	Development time (fast or normal)	300	9
Kwan & Rundle[94]	*Drosophila melanogaster*	Diet (access to food and water)	57	2
Leftwich et al.[95]†	*Ceratitis capitata*	Diet (starch or complex carbohydrates)	29	2
Magalhães et al.[96]	*Tetranychus urticae*	Diet (host plant: cucumber, pepper or tomato)	25	211
Maklakov et al.[97]	*Callosobruchus maculatus*	Age at reproduction (young or old)	15, 18	4
Meffert & Regan[98]	*Musca domestica*	Artificial selection on courtship traits	8	16
Miyatake & Shimizu[99]	*Zeugodacus cucurbitae*	Development time (fast or slow)	17, 38, 47, 48	13
Mooers et al.[100]	*Drosophila melanogaster*	Diet (regular or low pH)	21	51
Nash et al.[101]†	*Ceratitis capitata*	Diet (sucrose or starch)	60, 90	18
Rice[102]	*Drosophila melanogaster*	Female coevolution to males or not	40	2
Robinson et al.[103]	*Drosophila melanogaster*	Age at reproduction (young or old)	1500	30
Rodrigues et al.[104]	*Tetranychus urticae*	Female coevolution with *Wolbachia*-infected or uninfected males	12	48

Rova & Björklund[105]	*Callosobruchus maculatus*	Diet (blacked-eyed beans or mung beans)	9	2
Rova & Björklund[106]‡	*Callosobruchus maculatus*	Diet (blacked-eyed beans or mung beans)	10	19
Rova & Björklund[107]‡	*Callosobruchus maculatus*	Diet (blacked-eyed beans or mung beans)	11, 12, 13, 14, 15, 16, 17	308
Rundle[32]	*Drosophila pseudoobscura*	Diet and temperature (banana at 21 or cornmeal at 25)	15	58
Shenoi et al.[72]	*Drosophila melanogaster*	High or low larval density	118	8
Stojković et al.[108]	*Acanthoscelides obtectus*	Age at reproduction (young or old)	155, 222	12
Stojković et al.[73]	*Acanthoscelides obtectus*	Diet (host plant: bean or chick-pea)	10	8
Villa et al.[109]	*Columbicola columbae*	Pigeon host (giant runt or feral pigeon)	60	2

Supplementary Table 4 | Results from publication bias and heterogeneity analyses performed using two statistical packages on the dataset without including any covariates.

		Model Parameters		Heterogeneity (%)			
	**R package**	**int**	**slope**	$I^2_{species}$	$I^2_{phylogeny}$	$I^2_{research}$	$I^2_{residual}$
Egger's	MCMCglmm	0.086	0.094 (p = 0.06)	1	1	10	88
	metafor	0.071	0.820 (p < 0.001)	0	0	18	81
Time-lag	MCMCglmm	–0.764	0.001 (p = 0.84)	2	1	10	87
	metafor	0.165	-0.136 (p = 0.99)	0	0	0	0
Heterogeneity	MCMCglmm	0.103	-	2	1	7	90
	metafor	0.083	-	0	0	18	81

Supplementary Table 5 | Results of the main model from "metafor". Table of coefficients for the main model, including the interaction between the barrier type and whether a common garden generation was used. Tests used were two-sided.

	**estimate**	**se**	**t-value**	**p-value**	**95% CIs**
Intercept	–0.058	0.071	–0.818	0.445	[–0.232, 0.116]
Between treatments	0.072	0.014	5.024	< 0.001	[0.044, 0.101]
Pre-mating barrier	0.173	0.041	4.279	< 0.001	[0.094, 0.252]
Common garden present	0.023	0.062	0.377	0.707	[–0.098, –0.145]
Number of generations	–0.000	0.000	–0.216	0.829	[–0.001, 0.001]
Population-level data	0.052	0.043	1.196	0.232	[–0.033, 0.137]
Backtransformed	–0.025	0.044	–0.555	0.579	[–0.112, 0.062]
Founding population size (ln-transformed)	–0.001	0.007	–0.169	0.867	[–0.015, 0.013]
Pre-mating barrier : Common garden present	–0.130	0.061	–2.121	0.034	[–0.250, –0.010]
