# Supplementary material for: Meta-analysis reveals that phenotypic plasticity and divergent selection promote reproductive isolation during incipient speciation
Source: Nat Ecol Evol. 2025 May 7;9(5):833–44. doi: 10.1038/s41559-025-02687-7 (PMC12066359; doi:10.1038/s41559-025-02687-7)
Supplement: Supplementary file 2 — Reporting Summary [file 41559_2025_2687_MOESM2_ESM.pdf]

nature portfolio

Corresponding author(s): Benjamin JM Jarrett (b.jarrett@bangor.ac.uk)

Last updated by author(s): Mar 21, 2025

Reporting Summary

Nature Portfolio wishes to improve the reproducibility of the work that we publish. This form provides structure for consistency and transparency in reporting. For further information on Nature Portfolio policies, see our Editorial Policies and the Editorial Policy Checklist.

Statistics

For all statistical analyses, confirm that the following items are present in the figure legend, table legend, main text, or Methods section.

n/a	Confirmed	
☐	☒	The exact sample size (*n*) for each experimental group/condition, given as a discrete number and unit of measurement
☐	☒	A statement on whether measurements were taken from distinct samples or whether the same sample was measured repeatedly
☐	☒	The statistical test(s) used AND whether they are one- or two-sided<br>*Only common tests should be described solely by name; describe more complex techniques in the Methods section.*
☐	☒	A description of all covariates tested
☐	☒	A description of any assumptions or corrections, such as tests of normality and adjustment for multiple comparisons
☐	☒	A full description of the statistical parameters including central tendency (e.g. means) or other basic estimates (e.g. regression coefficient) AND variation (e.g. standard deviation) or associated estimates of uncertainty (e.g. confidence intervals)
☐	☒	For null hypothesis testing, the test statistic (e.g. *F*, *t*, *r*) with confidence intervals, effect sizes, degrees of freedom and *P* value noted<br>*Give P values as exact values whenever suitable.*
☐	☒	For Bayesian analysis, information on the choice of priors and Markov chain Monte Carlo settings
☐	☒	For hierarchical and complex designs, identification of the appropriate level for tests and full reporting of outcomes
☐	☒	Estimates of effect sizes (e.g. Cohen's *d*, Pearson's *r*), indicating how they were calculated

Our web collection on statistics for biologists contains articles on many of the points above.

Software and code

Policy information about availability of computer code

Data collection	Data were extracted from papers that successfully passed through the PRISMA checklist. Raw data was obtained from the supplementary information, from the authors, or means and errors were extracted from the images/tables using metaDigitise_1.0.1
Data analysis	R (4.2.2) was used for all analyses. Packages used for analysis were: metaAidR_0.0.0.9000, MCMCglmm_2.35, metafor_4.4.0, brms_2.18.0

For manuscripts utilizing custom algorithms or software that are central to the research but not yet described in published literature, software must be made available to editors and reviewers. We strongly encourage code deposition in a community repository (e.g. GitHub). See the Nature Portfolio guidelines for submitting code & software for further information.

Data

Policy information about availability of data

All manuscripts must include a data availability statement. This statement should provide the following information, where applicable:

- Accession codes, unique identifiers, or web links for publicly available datasets
- A description of any restrictions on data availability
- For clinical datasets or third party data, please ensure that the statement adheres to our policy

Data are available as supplementary information and on figshare (https://doi.org/10.6084/m9.figshare.27233727)

Research involving human participants, their data, or biological material

Policy information about studies with human participants or human data. See also policy information about sex, gender (identity/presentation), and sexual orientation and race, ethnicity and racism.

Reporting on sex and gender	NA
Reporting on race, ethnicity, or other socially relevant groupings	NA
Population characteristics	NA
Recruitment	NA
Ethics oversight	NA

Note that full information on the approval of the study protocol must also be provided in the manuscript.

Field-specific reporting

Please select the one below that is the best fit for your research. If you are not sure, read the appropriate sections before making your selection.

☐ Life sciences ☐ Behavioural & social sciences ☒ Ecological, evolutionary & environmental sciences

For a reference copy of the document with all sections, see nature.com/documents/nr-reporting-summary-flat.pdf

Ecological, evolutionary & environmental sciences study design

All studies must disclose on these points even when the disclosure is negative.

Study description	The study is a meta-analysis of estimates of reproductive isolation obtained in experimental speciation studies. A single population is split into replicates that experience the same (within treatment) or divergent (between treatment) environments. Reproductive isolation was estimated between populations within and between treatments to quantitatively assess ecological speciation at its onset.
Research sample	Data were obtained from papers searched for using a search string and filtered using a PRISMA flow diagram. The dataset compiled was 1723 effect sizes from 34 studies on 15 species.
Sampling strategy	Any relevant paper found in the two searches were kept for inclusion. If no estimate of reproductive isolation could be calculated (i.e., if there was no raw data or means with errors reported) the studies were excluded.
Data collection	One author, BJMJ, extracted all the data and calculated the appropriate effect sizes.
Timing and spatial scale	The search spanned the publication of Rice and Hostert (1993) in December 1993 to June 2023, when the final search was performed.
Data exclusions	No relevant data were excluded.
Reproducibility	All the code to extract the raw data from the filtered studies, as well as the code for the final meta-analysis, is included in the supplementary material.
Randomization	There was no randomisation as we only had the data that was available to us, either from means and standard errors published in the paper or via the raw data.
Blinding	Blinding was not possible for this study. BJMJ worked on extracting the data from each paper in turn.

Did the study involve field work? ☐ Yes ☒ No

Reporting for specific materials, systems and methods

We require information from authors about some types of materials, experimental systems and methods used in many studies. Here, indicate whether each material, system or method listed is relevant to your study. If you are not sure if a list item applies to your research, read the appropriate section before selecting a response.

Materials & experimental systems

n/a	Involved in the study
☒	☐ Antibodies
☒	☐ Eukaryotic cell lines
☒	☐ Palaeontology and archaeology
☒	☐ Animals and other organisms
☒	☐ Clinical data
☒	☐ Dual use research of concern
☒	☐ Plants

Methods

n/a	Involved in the study
☒	☐ ChIP-seq
☒	☐ Flow cytometry
☒	☐ MRI-based neuroimaging

Plants

Seed stocks	NA
Novel plant genotypes	NA
Authentication	NA
